# Electronic communication facilitates investigation of a highly dispersed foodborne outbreak: Salmonella on the superhighway.

**DOI:** 10.3201/eid0103.950306

**Published:** 1995

**Authors:** B E Mahon, D D Rohn, S R Pack, R V Tauxe

**Affiliations:** National Center for Infectious Diseases, Centers for Disease Control and Prevention, Atlanta, Georgia, USA.


### Electronic Communication Facilitates Investigation of a Highly Dispersed Foodborne Outbreak: *Salmonella* on the Superhighway

Widely dispersed foodborne disease outbreaks are an emerging public health problem (1-3). Increasing population mobility and the wide distribution of centrally produced foods mean that when an outbreak of foodborne disease occurs, the affected persons may be distributed across the country or even the world. Widely dispersed outbreaks challenge limited public health resources; they can be difficult to detect, labor-intensive, and time- consuming to investigate. We report the rapid, efficient investigation of a widely dispersed interstate outbreak through electronic communication between the possible patients and public health workers.

On August 4, 1994, a resident of a western state contacted the Centers for Disease Control and Prevention (CDC) regarding a possible outbreak of foodborne illness. On July 22, the day after returning from a conference in Baltimore that included attendees from all 50 states, he became ill with diarrhea, and *Salmonella* was isolated from his stool. He contacted four other conference attendees who had taken the same flight. One of these also had culture-confirmed *Salmonella* infection; a second, who was taking antibiotics for other reasons, had a diarrheal illness with a negative stool culture, and two had nonspecific diarrheal illnesses. Because of the possibility of a multistate outbreak involving the airline or the conference and affecting many people, we initiated a survey of conference attendees to determine the rate and correlates of diarrheal illness.

Traditionally, surveys of dispersed populations have been conducted either by telephone, requiring many person-hours of interviewing, or by mail, leading to many days' delay while questionnaires are distributed and returned. However, in this case, the organization that sponsored the conference had an internal electronic mail (e-mail) system. Each section of the organization, although not each person, had a computer that could receive e-mail messages. On August 5, 1994, the organization's central office e-mailed a questionnaire, developed in consultation with CDC, to all computers in the organization with instructions to organization staff to print and distribute the questionnaire to conference attendees. The questionnaire contained items regarding diarrheal illness, meal and flight exposures, and demographic information. Because the organization's e-mail system was only internal, responses could not be made to CDC by e-mail. Therefore, attendees were instructed to send their completed questionnaires to CDC by fax. By August 12 (7 days later), sufficient responses had been received to evaluate the flight and the conference as sources of an outbreak of salmonellosis (Figure 1).

Of 390 persons registered at the conference, 86 (22%) returned questionnaires by August 12. A questionnaire was returned by the index patient who made the initial call to CDC but not by the four other persons he contacted who were passengers on the same flight. Six (7%) of the 86 respondents reported having diarrhea (three or more loose stools in a 24-hour period) during the period beginning 12 hours after the conference started and ending 5 days after the conference ended (July 20 to 26). Among questionnaire respondents, only the index patient was diagnosed with salmonellosis. Three respondents had taken the initially suspect flight. Illness was not associated with taking the same flight as the index patient ($p = 0.20$, Fisher's Exact Test, 2-tailed).

To further investigate the reports of diarrhea, we interviewed the six persons who reported diarrheal illness by questionnaire, as well as the four persons initially contacted by the index patient who had not completed questionnaires. This group included the two persons with known *Salmonella* infection, of whom one had completed a questionnaire and one had not. Seven of the other eight persons had mild, nonspecific symptoms of less than 2 days' duration; the onset dates of their illnesses spanned a 5-day period, and none sought medical attention. Because few conference attendees or flight passengers became ill with symptoms suggestive of salmonellosis during a likely period, we thought that an airplane- or conference-associated outbreak was improbable.

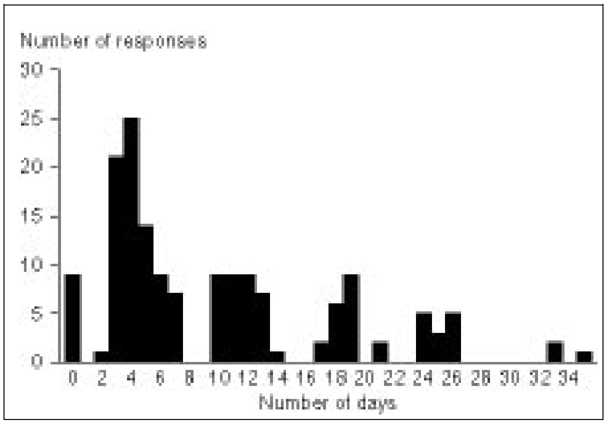

Figure 1. Number of days from questionnaire distribution by e-mail to questionnaire return by fax (n=156). Day 0 is Friday, August 5, 1994. Range, 0 to 35 days; median = 6 days.

By September 9, questionnaires were returned by 156 (40%) of the conference attendees. No additional cases of diarrhea were reported, confirming our initial conclusion that the *Salmonella* infections were not associated with the flight or the conference.

The *Salmonella* isolates were identified at CDC as *Salmonella* serotype Norwich, of the *Salmonella* serogroup C1. *S*. Norwich is rare; in 1993 and 1994, respectively, 63 and 102 isolates of this serotype were reported to the Public Health Laboratory Information System (PHLIS), a nationwide electronic laboratory-based surveillance system that collects and summarizes data on isolates from state public health laboratories (4). Because infection with *S*. Norwich is so uncommon, it still seemed likely that the two infections could have a common source, such as a restaurant.

Subsequent investigation focused on meals that the two persons with salmonellosis shared outside the conference and ultimately revealed the source, a restaurant in Baltimore. In late July 1994, the Maryland Department of Health and Mental Hygiene received reports that *Salmonella*, serogroup C1, had been isolated from five other persons who visited Baltimore around the time of the conference. Two persons from one family had driven to Baltimore on July 17, eaten only at one restaurant, then returned to their home state of Pennsylvania. Three persons in a second family, from a different part of Pennsylvania, ate at the same Baltimore restaurant on July 21 during a vacation trip. The *Salmonella* isolates from members of both families were initially misidentified as other serogroup C1 serotypes. They were retested because of this outbreak and were confirmed as *S*. Norwich. The two conference attendees with *S*. Norwich infection also ate at the implicated restaurant on July 21. No single menu item had been eaten by all ill persons. In response to a complaint by the first family, the restaurant had been inspected by the local health department; multiple violations of food safety regulations were found. *S*. Norwich was isolated from a stool specimen from an employee who reported a diarrheal illness beginning on July 22 and who ate the restaurant's food. In the month following the inspection of Restaurant A and subsequent corrective action, no further cases of *S*. Norwich were reported to PHLIS from Maryland or Pennsylvania.

E-mail can expedite questionnaire distribution, especially when the population of interest is on one network. The computer system used to send the e-mail message in this outbreak was not linked to individual conference attendees; therefore, we could not evaluate the rates at which individual attendees obtained and responded to the message. If we had been able to reach attendees directly, our response rate may have been higher, and we would have been able to send additional messages to nonresponders. In the future, when outbreaks occur among persons accessible by e-mail, it may be possible to evaluate strategies to improve response rate and to compare the effectiveness of the delivery of questionnaires by e-mail and by more traditional means.

This outbreak illustrates the usefulness of rapid electronic communication in a public health setting. Isolation of a rare *Salmonella* serotype and national electronic reporting to PHLIS assisted in the detection and investigation of a widely dispersed multistate outbreak of salmonellosis. Without the national *Salmonella* serotyping system, the outbreak would not have been recognized. Questionnaires were distributed rapidly by e-mail; the utility of this method is likely to increase as more people become accessible by e-mail. Fax provided a means for respondents to return questionnaires quickly. Continued on-line analysis of surveillance data with PHLIS confirmed that the outbreak was controlled. Rapid communication between public health workers in Maryland and Pennsylvania and at CDC was also essential. The usefulness of electronic communication is not limited to outbreak investigation. New technologies will undoubtedly continue to be useful in addressing emerging public health problems.

**Barbara E. Mahon,* Dale D. Rohn,† Sheila R. Pack,‡ Robert V. Tauxe***

*National Center for Infectious Diseases, Centers for Disease Control and Prevention, Atlanta, Georgia, USA; †Epidemiology and Disease Control Program, Maryland Department of Health and Mental Hygiene, Baltimore, Maryland, USA; ‡Division of Acute Communicable Diseases (now in Division of HIV Seroepidemiology), Baltimore City Health Department, Baltimore, Maryland, USA

### References

1. Tauxe RV. *Salmonella*: a postmodern pathogen. J Food Protection 1991;54:563-8.
2. Hedberg CW, MacDonald KL, Osterholm MT. Changing epidemiology of food-borne disease: a Minnesota perspective. Clin Infect Dis 1994;18:671-82.
3. Hedberg CW, Levine WC, White KE, Carlson RH, Winsor DK, Cameron DN, et al. An international foodborne outbreak of shigellosis associated with a commercial airline. JAMA 1992;268:3208-12.
4. Bean NH, Martin SM, Bradford H. PHLIS: an electronic system for reporting public health data from remote sites. Am J Public Health 1992;82:1273-6.
